# Glycemic assessment in diabetic patients with secondary polycytheamia: limitations of HbA1c and the potential role of fructosamine—a prospective comparative study

**DOI:** 10.1007/s00592-026-02735-z

**Published:** 2026-06-22

**Authors:** Sertas Erarslan, Turkan P. Kilit, Aycan Acet

**Affiliations:** Faculty of Medicine, Department of Internal Medicine, Kutahya Health Sciences University, Bahcelievler Street, Kütahya, 43000 Türkiye

**Keywords:** HbA1c, Fructosamine, Polycythaemia, Glycaemic assessment, Diabetes mellitus

## Abstract

**Objective:**

This study aimed to evaluate the reliability of haemoglobin A1c (HbA1c) for assessing glycaemic status in patients with type 2 diabetes mellitus (T2DM) and secondary polycythaemia, and to determine whether fructosamine serves as a more reliable complementary glycaemic marker in this population.

**Methods:**

In this prospective observational study, patients with T2DM and secondary polycythaemia (*n* = 62) and T2DM controls with normal haematological indices (*n* = 40) were enrolled. HbA1c- and fructosamine-derived estimated mean plasma glucose (eMPG) were compared with structured home capillary blood glucose (CBG; 7 measurements/day × 5 days). Bland-Altman analysis and multivariate linear regression (adjusting for age, sex, diabetes duration, BMI, smoking, fasting glucose, haemoglobin, haematocrit) were performed.

**Results:**

Fasting plasma glucose and HbA1c were similar between groups (*p* = 0.352 and *p* = 0.816), while fructosamine (456.53 ± 90.18 vs. 404.18 ± 97.63 µmol/L; *p* = 0.001) and Home-CBG (207.23 ± 49.18 vs. 181.98 ± 55.31 mg/dL; *p* = 0.002) were significantly higher in the polycythaemia group. Bland-Altman analysis confirmed a systematic negative bias of HbA1c-derived eMPG (mean bias: −23.73 mg/dL; 95% LoA: −45.97 to − 1.48). On multivariate regression, polycythaemia group assignment was the strongest independent association with HbA1c underestimation (β=−16.80 mg/dL; 95% CI: −25.53 to − 8.07; *p* < 0.001). Fructosamine-derived eMPG showed substantially better agreement with Home-CBG (bias: +7.68 mg/dL). Overall, 60% of polycythaemia patients would have been assigned to a different glycaemic control category based on fructosamine versus HbA1c.

**Conclusion:**

HbA1c systematically underestimates glycaemic status in patients with polycythaemia and may lead to clinically relevant misclassification. In contrast, fructosamine appears to provide a more reliable estimate of glycaemic control in this population.

**Supplementary Information:**

The online version contains supplementary material available at https://doi.org/10.1007/s00592-026-02735-z.

## Introduction

Diabetes mellitus (DM) is a chronic metabolic disorder characterized by impaired glycaemic regulation and a rapidly increasing global prevalence. Achieving and maintaining optimal glycaemic control is essential for preventing microvascular and macrovascular complications [1]. Glycated hemoglobin (HbA1c) is the most widely used parameter for assessing long-term glycaemic status and reflects average plasma glucose levels over approximately 8–12 weeks [2, 3].

HbA1c is formed through the non-enzymatic glycation of hemoglobin within erythrocytes and is therefore influenced by erythrocyte lifespan and turnover. Conditions that alter erythrocyte kinetics, including hemoglobinopathies, anemia, and various hematologic disorders, may lead to misleading HbA1c values independent of actual glycaemic status [4, 5]. Both shortened and prolonged erythrocyte survival have been shown to significantly affect HbA1c levels, potentially leading to underestimation or overestimation of glycaemic control.

In particular, a predominance of younger erythrocytes due to increased turnover may result in lower HbA1c levels despite sustained hyperglycaemia.

Polycythaemia is characterised by an increased circulating erythrocyte mass and may be classified as primary or secondary depending on the underlying aetiology [6]. Secondary polycythaemia most commonly arises from chronic hypoxic stimuli -in particular, tobacco smoking and high-altitude residence- which promote erythropoietin-driven erythrocytosis. In polycythaemia vera, erythrocyte lifespan has been shown to be shortened [7]; however, erythrocyte dynamics in secondary polycythaemia are less clearly defined. Increased erythropoiesis, altered age distribution of circulating erythrocytes, and changes in plasma volume may all influence HbA1c measurements. Haemoglobin concentration itself has also been shown to independently affect HbA1c in non-anaemic individuals [19]. Whether these alterations lead to a systematic bias in HbA1c-derived glycaemic estimates in secondary polycythaemia remains uncertain.

In clinical situations where HbA1c may be unreliable, alternative glycaemic markers are recommended. Fructosamine, which reflects glycation of circulating plasma proteins, especially primarily albumin, provides an estimate of average glucose levels over the preceding 2–4 weeks and is not directly affected by erythrocyte lifespan [8, 9]. Previous studies have suggested that fructosamine may be useful in conditions where HbA1c interpretation is limited and may also be associated with diabetic complications [10].

Although the limitations of HbA1c in conditions affecting erythrocyte turnover are well recognised, prospective evidence specifically addressing glycaemic assessment in diabetic patients with secondary polycythaemia remains scarce. Moreover, most available studies rely on group-level comparisons; within-subject data are lacking. The present study was conducted at Kütahya City (~ 969 m above sea level), a setting where both altitude- and smoking-related erythrocytosis are prevalent, and aimed to evaluate the concordance of HbA1c, fructosamine, and home capillary glucose measurements in patients with type 2 diabetes and secondary polycythaemia, and to investigate whether fructosamine may provide additional clinical value when HbA1c appears discordant.

## Materials and methods

### Study design and subjects

This prospective observational study was conducted between 01 September 2025 and 30 November 2025 in the Internal Medicine outpatient clinics of Kütahya City Hospital, Kütahya, Turkey. Kütahya is situated at approximately 969 m above sea level, a geographic context directly relevant to the etiology of secondary polycythaemia in this cohort, as moderate-altitude hypoxic stimulation of erythropoietin secretion is a well-established cause of erythrocytosis [25, 26]. A total of 102 participants were enrolled: 62 adult patients with type 2 DM and secondary polycythaemia, and 40 adult patients with type 2 DM without polycythaemia (control group).

Polycythaemia was defined as a hemoglobin (Hgb) level > 16.5 g/dL and/or hematocrit (Hct) > 49% in males, and Hgb > 16 g/dL and/or Hct > 48% in females [11].

Patients identified with polycythaemia were evaluated in consultation with the haematology department to exclude polycythaemia vera. All remaining participants were assessed for potential secondary causes of erythrocytosis. Only patients with secondary polycythaemia were included; the etiology was further characterised in each case as described below. Patients with other identifiable secondary causes -including chronic pulmonary diseases unrelated to smoking, obstructive sleep apnoea, cyanotic congenital heart disease, or erythropoietin-producing tumours- were excluded.

### Etiology of secondary polycythaemia in the study cohort

Among the 62 patients in the polycythaemia group, 44 (71.0%) had an active or former smoking history, representing the predominant identified contributing factor. The remaining 18 patients (29.0%) had no smoking history; in this subgroup, chronic altitude exposure at approximately 969 m above sea level is considered the most probable primary aetiology, given the geographic setting and the exclusion of other secondary causes. It is important to note that altitude-related erythrocytosis in this subgroup is inferred from the clinical context rather than directly documented through formal hypoxia assessment (e.g. erythropoietin levels, pulse oximetry, or sleep apnoea screening); this inferential classification represents an acknowledged limitation of the present study. COPD was documented in only 1 patient (1.6%).

### Inclusion criteria

Patients aged ≥ 18 years with a confirmed diagnosis of type 2 DM and polycythaemia, who had the cognitive and physical ability to perform home glucose monitoring, were included in the study.

### Exclusion criteria

Patients with anaemia (Hgb < 13 g/dL in men, < 12 g/dL in women), haematological malignancies, haemoglobinopathies, chronic liver disease, and chronic kidney disease (CKD) were excluded. CKD was specifically excluded because it independently impairs HbA1c reliability through mechanisms distinct from polycythaemia -including erythropoietin deficiency-related anaemia and uraemia-associated altered protein glycation- which would have introduced uncontrolled confounding. Polycythaemia vera, obstructive sleep apnoea, cyanotic congenital heart disease, and erythropoietin-producing tumours were additional exclusion criteria.

The control group consisted of patients with type 2 DM whose Hgb and Hct values were within normal limits (Hgb ≤ 16 g/dL and Hct ≤ 45% in men; Hgb ≤ 15 g/dL and Hct ≤ 42% in women).

### Study data

Demographic and anthropometric data, including age, sex, body mass index (BMI), and waist circumference, were recorded for all participants. Laboratory values obtained during routine follow-up visits included fasting plasma glucose, HbA1c [measured using high-performance liquid chromatography (HPLC)], complete blood count parameters (WBC, neutrophils, lymphocytes, Hgb, Hct, MCV, PLT, MPV), lipid profile (total cholesterol, HDL cholesterol, LDL cholesterol, triglycerides), blood urea nitrogen (BUN), creatinine, and uric acid levels, all retrieved from hospital records.

For fructosamine analysis, blood samples were obtained from consenting patients (provided that vitamin C supplements had not been used within the previous 72 h), stored at − 80 °C, and analysed using a colorimetric method.

### Glucose monitoring

Continuous glucose monitoring (CGM) was not employed due to accessibility and cost constraints; structured capillary blood glucose (CBG) measurements were used as a pragmatic real-world reference comparator. It is acknowledged that this approach does not constitute a gold standard equivalent to CGM, and this limitation is discussed below. All participants were trained to perform home glucose monitoring seven times daily (fasting; 2 h post-breakfast; pre-lunch; 2 h post-lunch; pre-dinner; 2 h post-dinner; bedtime) for five consecutive days (35 readings/participant). The mean (± SD) of all readings constituted the Home-CBG.

### eMPG calculation

HbA1c-derived estimated mean plasma glucose [eMPG (HbA1c)] was calculated using the equation proposed by Nathan et al.: eMPG (mg/dL) = (28.7 × HbA1c) − 46.7 [2].

Fructosamine-derived estimated mean plasma glucose [eMPG (fructosamine)] was calculated using the equation proposed by Andrade et al.: eMPG (mg/dL) = 0.5157 × fructosamine (µmol/L) − 20 [9].

For each participant, within-subject differences between estimated and measured glucose were calculated as Δ = eMPG − CBG, and these values were used to assess agreement between methods.

### Statistical analysis

Statistical analyses were performed using IBM SPSS version 22.0. Data distribution was assessed using histograms, skewness/kurtosis values, and the Kolmogorov–Smirnov test. Continuous variables are expressed as mean ± SD; categorical variables as n (%). Within-subject comparisons between eMPG and CBG were performed using the paired samples t-test. Between-group comparisons of Δ values were conducted using the independent samples t-test. Categorical variables were compared using the chi-square test. Results are presented with 95% confidence intervals (CI). Agreement between glycaemic methods was assessed by Bland-Altman analysis, reporting mean bias and 95% limits of agreement (LoA = mean bias ± 1.96 SD). Multivariate linear regression was performed with Δ(HbA1c) [= eMPG(HbA1c) − Home-CBG] as the outcome variable; covariates included polycythaemia group assignment, age, sex, diabetes duration, BMI, smoking status, fasting plasma glucose, haemoglobin, and haematocrit. A post-hoc power analysis confirmed that with *n* = 62, observed bias − 23.73 mg/dL, SD 11.35 mg/dL, and α = 0.05, statistical power exceeded 99.9%. A p-value < 0.05 was considered statistically significant throughout.

## Results

Baseline demographic and clinical characteristics are presented in Table 1. The control group was significantly older than the polycythemia group (62.38 ± 7.58 vs. 55.50 ± 10.26 years; *p* = 0.001). The proportion of male participants was significantly higher in the polycythemia group (81.0% vs. 30.0%; *p* < 0.001). BMI and diabetes duration were comparable between groups (*p* > 0.05). Comorbidities, including hypertension, hyperlipidemia, and coronary artery disease, did not differ significantly between groups. Antidiabetic treatment regimens showed no significant between-group differences for any medication class. Notably, SGLT2 inhibitor use -which is known to raise hematocrit- was comparable between groups (71.0% vs. 60.0%; *p* = 0.351), arguing against SGLT2-mediated erythrocytosis as a confounding explanation for the observed discordance.

Plasma glucose levels are summarized in Table 2. Fasting plasma glucose and HbA1c levels were similar between the two groups (*p* = 0.352 and *p* = 0.816, respectively). However, fructosamine levels were significantly higher in the polycythemia group (456.53 ± 90.18 µmol/L vs. 404.18 ± 97.63 µmol/L, *p* = 0.001). Similarly, fructosamine-derived eMPG and home capillary blood glucose (CBG) levels were significantly higher in the polycythemia group (*p* = 0.001 and *p* = 0.002, respectively).

Within-group comparisons of glucose estimation methods are presented in Table 3. In the polycythemia group, significant differences were observed among HbA1c-derived eMPG, fructosamine-derived eMPG, and home CBG measurements (*p* < 0.001), whereas no significant differences were observed in the control group (*p* = 0.110).

**Fig. 1:**
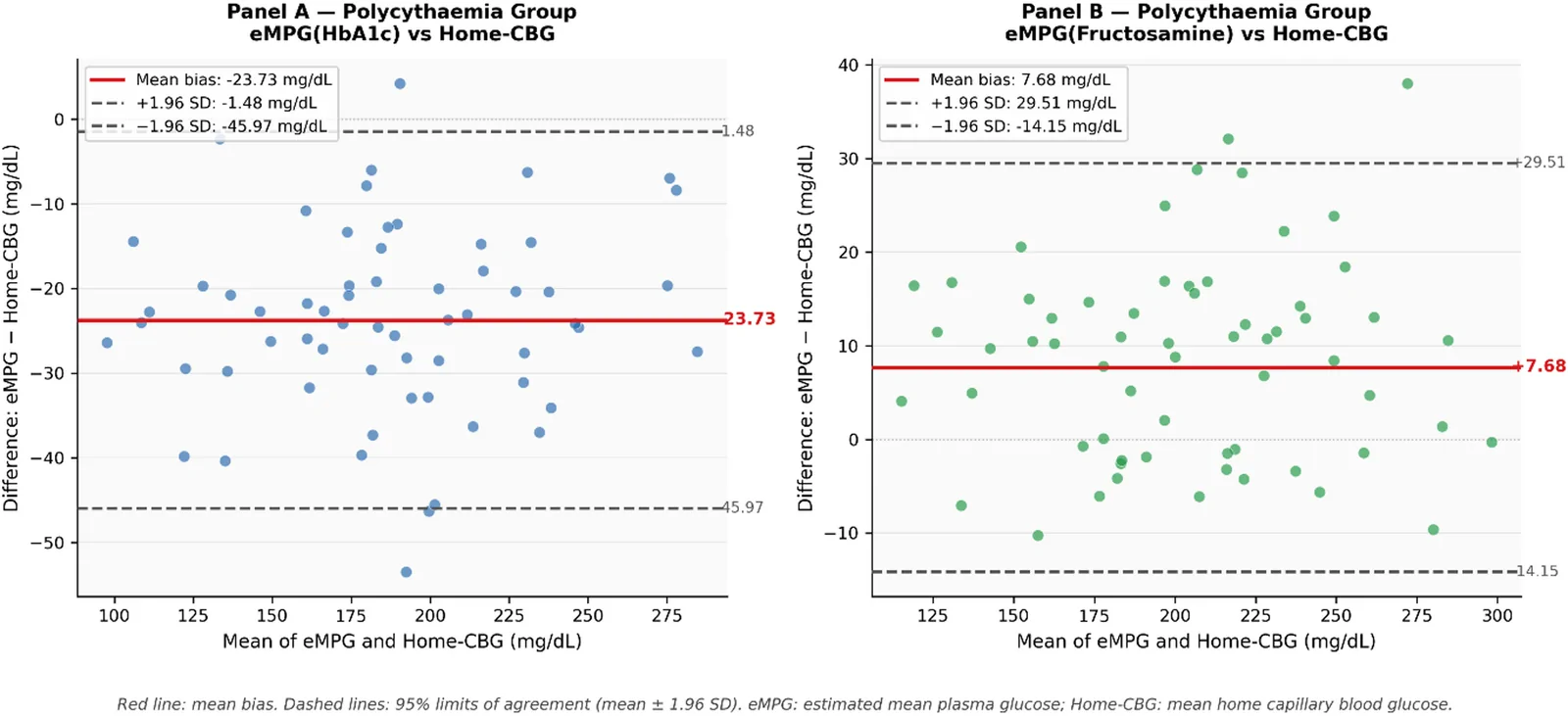
Bland-Altman agreement plots for eMPG(HbA1c) and eMPG(Fructosamine) versus Home-CBG, displayed separately for the polycythemia. Red line: mean bias; dashed lines: 95% limits of agreement (mean ± 1.96 SD). **A** (polycythemia, HbA1c): mean bias − 23.73 mg/dL (LoA: −45.97 to − 1.48), demonstrating systematic underestimation. **B** (polycythemia, fructosamine): bias + 7.68 mg/dL (LoA: −14.15 to + 29.51), indicating acceptable agreement. *LoA* limits of agreement; *eMPG* estimated mean plasma glucose; *Home-CBG* mean home capillary blood glucose

Bland-Altman analysis (Table 4; Fig. 1) confirmed a systematic negative bias of eMPG(HbA1c) versus Home-CBG in the polycythemia group (mean bias: −23.73 mg/dL; 95% LoA: −45.97 to − 1.48), indicating consistent underestimation of real-life glycemia. In the same group, eMPG(fructosamine) showed substantially better agreement (bias: +7.68 mg/dL; 95% LoA: −14.15 to + 29.51). In the control group, both eMPG(HbA1c) (bias: +0.23 mg/dL; LoA: −16.37 to + 16.82) and eMPG(fructosamine) (bias: +3.77 mg/dL; LoA: −7.50 to + 15.05) showed narrow, clinically acceptable limits of agreement.

Multivariate linear regression results are presented in Table 5. After full adjustment for age, sex, diabetes duration, BMI, smoking status, fasting glucose, haemoglobin, and haematocrit, polycythaemia group assignment was independently associated with HbA1c underestimation (β=−16.80 mg/dL; 95% CI: −25.53 to − 8.07; *p* < 0.001). Diabetes duration showed borderline independent significance (β=+0.38; 95% CI: +0.07 to + 0.68; *p* = 0.015). All other covariates, including haemoglobin and haematocrit, did not reach independent significance (*p* > 0.05). The model explained 62.2% of the variance in HbA1c bias (R²=0.622, adjusted R²=0.585; overall F-test *p* < 0.001).

Glycaemic reclassification analysis showed that eMPG(HbA1c) was lower than Home-CBG in 100% (62/62) of patients with polycythaemia. A discordance exceeding 20 mg/dL was observed in 63% (39/62) and exceeding 30 mg/dL in 26% (16/62). Overall, 60% (37/62) would have been assigned to a different glycaemic control category by eMPG(fructosamine) versus eMPG(HbA1c). These findings suggest that clinically meaningful glycaemic misclassification may occur when HbA1c is used as the sole glycaemic marker in this population, and warrant consideration in clinical decision-making pending outcome-based validation.

Other laboratory values are presented in Supplementary Table 6. Hemoglobin, hematocrit, and red blood cell counts were significantly higher in the polycythemia group (*p* = 0.001), confirming the expected hematological profile. Other biochemical and hematological parameters were comparable between groups.

## Discussion

This prospective observational study demonstrates that HbA1c systematically underestimates real-life glycaemia in patients with T2DM and secondary polycythaemia, and that this effect persists after full multivariate adjustment for potential confounders, a finding that extends beyond group-level comparisons to direct within-subject evidence of measurement bias. The magnitude of underestimation is clinically meaningful: it was sufficient to cause glycaemic misclassification in 60% of affected patients, a reclassification rate with direct implications for treatment decisions. In contrast, fructosamine showed substantially better agreement with real-life glucose measurements, and the smaller deviation observed is unlikely to be clinically significant.

HbA1c reflects average glycaemic exposure over approximately 8–12 weeks and is influenced by erythrocyte lifespan [2, 3]. Conditions affecting erythrocyte turnover may produce misleading HbA1c values. Several factors — including haemoglobin concentration, age, medications, and comorbidities—may also affect HbA1c levels [12]. Higgins demonstrated that variability in erythrocyte lifespan significantly influences HbA1c independently of glycaemia [13]. Prolonged erythrocyte survival results in falsely elevated HbA1c [14], whereas shortened survival leads to falsely low values [15–17]. Similarly, haemoglobinopathies and assay-related factors may further affect HbA1c accuracy [18]. Bae et al. demonstrated that HbA1c values may vary with haemoglobin levels even in non-anaemic individuals [19]. In this context, increased erythropoiesis driven by chronic hypoxic stimuli may result in a higher proportion of younger erythrocytes with reduced glycation exposure time, thereby lowering HbA1c despite sustained hyperglycaemia. This mechanistic hypothesis is consistent with available literature; however, erythrocyte lifespan was not directly measured in this study and this interpretation remains hypothetical.

In clinical conditions where HbA1c measurement may be affected, the use of alternative glycaemic markers is recommended [1, 20]. Fructosamine reflects glycation of circulating plasma proteins, primarily albumin, and provides an estimate of glycaemic control over the preceding 2–4 weeks [8, 9]. Its independence from erythrocyte lifespan makes it particularly advantageous in hematologic conditions. Several studies have demonstrated the reliability of fructosamine as a glycaemic marker. Chandran et al. reported a significant correlation between fructosamine and HbA1c [21]. Lee emphasized the importance of alternative biomarkers when HbA1c interpretation is unreliable [22]. In surgical patients, fructosamine has been shown to be a reliable short-term glycaemic marker and may more accurately reflect short-term glycaemic status compared to HbA1c [23]. These findings support the reliability of fructosamine. In our study, in the non-polycythaemic group, glucose values estimated from HbA1c, fructosamine-derived glucose, and mean home capillary blood glucose measurements were consistent with each other, further supporting the reliability of fructosamine and aligning with the existing literature.

Despite similar HbA1c levels between groups, fructosamine levels, fructosamine-derived glucose values, and mean home capillary blood glucose measurements were higher in the polycythaemic group. HbA1c-derived glucose values were lower than home capillary blood glucose measurements, whereas fructosamine-derived values were consistent with them. The available literature evaluating the impact of polycythaemia on HbA1c measurement in diabetic patients is limited. Borade et al. suggested that fructosamine may provide a more accurate assessment than HbA1c in this population [24]; however, their study was limited by a small sample size and lacked direct glucose validation. In contrast, our study incorporated multiple glycaemic indicators including real-life home glucose measurements. Increased erythropoiesis, a higher proportion of younger erythrocytes, shortened glycation exposure time, and increased erythrocyte mass in secondary polycythaemia are hypothesised to explain the lower HbA1c levels observed; however, as erythrocyte lifespan was not directly measured, this mechanism remains a hypothesis rather than an established finding.

The geographic context of this study warrants consideration. Kütahya is situated at approximately 969 m above sea level, a moderate altitude associated with a well-established hypoxic erythropoietic stimulus [25, 26]. Among the non-smoking subgroup (29%), chronic altitude exposure represents the most plausible primary aetiology after exclusion of other secondary causes. The co-existence of smoking-related and altitude-related erythrocytosis in a single cohort reflects real-world clinical practice in similar geographic settings and strengthens external validity for clinicians practising in moderate-altitude populations.

The multivariate regression analysis provides an important advance beyond group-level comparisons. Polycythaemia group assignment was independently associated with HbA1c underestimation (β=−16.80 mg/dL; *p* < 0.001) after adjustment for haemoglobin and haematocrit — neither of which reached independent significance — suggesting that the relevant mechanism is not simply haemoglobin concentration per se but rather the underlying erythropoietic state. Comparable SGLT2 inhibitor use between groups (71.0% vs. 60.0%; *p* = 0.351) further argues against SGLT2-mediated haematocrit elevation as a confounding explanation. The independent significance of diabetes duration (β=+0.38; *p* = 0.015) may reflect accumulating erythrocyte exposure to chronic hyperglycaemia, though this requires confirmation.

From a clinical perspective, reliance solely on HbA1c in diabetic patients with secondary polycythaemia may lead to misinterpretation of glycaemic control. When discordance is observed between HbA1c and clinical findings, fructosamine may be used as a complementary biomarker to assess glycaemic status. These findings highlight the importance of considering alternative biomarkers in specific clinical settings, particularly in geographic areas where altitude- and smoking-related erythrocytosis are prevalent.

This study has several limitations. It was conducted in a single centre with a relatively small sample size. Although polycythaemia vera and other identifiable secondary causes were clinically excluded, erythropoietin levels, oxygen saturation, and sleep apnoea screening were not systematically performed; and among non-smoking patients, altitude-related erythrocytosis is inferred rather than directly documented — a limitation that should be considered when interpreting the aetiological distribution. Fructosamine levels may be influenced by protein metabolism; serum albumin was not measured, precluding direct adjustment for its potential influence on fructosamine values. Continuous glucose monitoring was not employed; home capillary blood glucose represents a pragmatic real-world comparator rather than a reference standard, and findings should be interpreted accordingly. Glucometer device standardisation across patients was not performed. The predominantly male, high-smoking-prevalence cohort studied at moderate altitude limits generalisability to female patients and non-smoking aetiologies at sea level. Reticulocyte count and erythropoietin levels were not measured; future studies incorporating these parameters alongside direct erythrocyte lifespan assessment would allow mechanistic hypotheses to be tested directly. However, the prospective design and integration of biomarker data with real-life glucose measurements represent important strengths.

**Table 1:** Demographic and general characteristics of the study participants

	Polycythaemia group (*N* = 62)	Control group (*N* = 40)	*P*-value
Age (years) (mean ± SD)	55.5 ± 10.26	62.38 ± 7.58	**< 0.001**
Height (cm) (mean ± SD)	168.42 ± 8.5	162.3 ± 9.7	**0.001**
BMI, kg/m^2^ (mean ± SD)	29.4 ± 4.53	30.44 ± 3.74	0.165
DM-duration (mean ± SD)	8.37 ± 7.17	9.8 ± 7.08	0.188
Gender			**0.001**
Male [N (%)]	50 (80.64%)	12 (30%)	
Female [N (%)]	12 (19.36%)	28 (70%)	
Smoke			**< 0.001**
Smoker [N (%)]	44 (71.0%)	11 (27.5%)	
No-smoker [N(%)]	18 (29.0%)	29 (72.5%)	
Co-morbidities			
HT [N (%)]	28 (45.16%)	26 (65%)	**0.05**
HL [N (%)]	36 (58.06%)	23 (57.5%)	0.56
COPD [N(%)]	1 (1.61%)	0 (0%)	0.61
CAD [N(%)]	11 (17.74%)	4 (10%)	0.22
Drug use			
Basal insulin [N (%)]	25 (40.3%)	13 (32.5%)	0.556
Rapid-acting insulin [N (%)]	16 (25.8%)	9 (22.5%)	0.886
Metformin [N (%)]	40 (64.5%)	28 (70%)	0.720
Sulphonylurea [N (%)]	13 (21%)	9 (22.5%)	1.000
SGLT-2 inhibitor [N (%)]	44 (71%)	24 (60%)	0.351
DPP-4 inhibitor [N (%)]	28 (45.2)	22 (55%)	0.443

Bold values ​​indicate statistical significance (p < 0.05)

The Mann-Whitney U test was used for numerical data, and the Chi-square test for categorical data

*BMI* body mass index, *DM duration* duration of diabetes diagnosis, *HT* hypertension, *HL* hyperlipidemia, *COPD* chronic obstructive pulmonary disease, *CAD* coronary artery disease, *SGLT2* sodium-glucose cotransporter-2; *DPP-4* dipeptidyl peptidase-4

**Table 2:** Plasma glucose levels

	Polycythaemia group (*N*: 62)	Control group (*N*:40)	*P*-value
	Mean ± SD	Mean ± SD	
FPG (mg/dL)	166.59 ± 60.97	156.70 ± 64.65	0.352
HbA1c	8.02 ± 1.78	8.00 ± 1.92	0.816
*Fructosamine (µmol/L)*	*456.53 ± 90.18*	*404.18 ± 97.63*	**0.001**
eMPG(HbA1c)	184.11 ± 51.12	183.46 ± 55.11	0.816
*eMPG(fructosamine)*	215.43 ± 46.50	188.43 ± 50.35	**0.001**
*Home-CBG*	207.23 ± 49.18	181.98 ± 55.31	**0.002**

Bold values ​​indicate statistical significance (p < 0.05)

Mann-Whitney U test

*FPG* fasting plasma glucose, *HbA1c-derived eMPG* HbA1c-derived estimated mean plasma glucose level, *Fructosamine-derived eMPG* fructosamine-derived estimated mean plasma glucose level, *Home-CGM* mean capillary glucose levels measured at home

**Table 3:** Estimated plasma glucose levels according to HbA1c and fructosamine measurements

	*N*	eMPG (HbA1c)		eMPG (Fructosamine)		Home-CBG		*P* value
		Mean	±SD	Mean	±SD	Mean	±SD	
Polycythemia group	62	183.51	51.32	215.19	46.85	207.23	49.18	**< 0.001**
Control group	40	181.98	55.31	185.65	50.95	182.01	55.47	0.110

Bold values ​​indicate statistical significance (p < 0.05)

Friedmann testi

*HbA1c-derived eMPG* HbA1c-derived estimated mean plasma glucose level, *Fructosamine-derived eMPG* fructosamine-derived estimated mean plasma glucose level, *Home-CGM* mean capillary glucose measurement at home

**Table 4:** Bland-Altman agreement analysis

Group	Comparison	*N*	Mean Bias (mg/dL)	95% LoA (mg/dL)
Polycythaemia	eMPG (HbA1c) vs. Home-CBG	62	**−23.73**	−45.97 to − 1.48
Polycythaemia	eMPG (Fructosamine) vs. Home-CBG	62	+ 7.68	−14.15 to + 29.51
Control	eMPG(HbA1c) vs. Home-CBG	40	+ 0.23	−16.37 to + 16.82
Control	eMPG(Fructosamine) vs. Home-CBG	40	+ 3.77	−7.50 to + 15.05

Highlighted rows indicate the clinically significant systematic underestimation in the polycythaemia group

*LoA* limits of agreement (mean bias ± 1.96 SD of differences). *eMPG* estimated mean plasma glucose; *Home-CBG* mean home capillary blood glucose

**Table 5:** Multivariate linear regression: independent predictors of HbA1c underestimation

Variable	β (mg/dL)	SE	95% CI	*p*-value
*Polycythemia group (vs. control)*	−16.80	4.40	−25.53 to − 8.07	< 0.001*
Age (years)	+ 0.02	0.13	−0.23 to + 0.27	0.864
Sex (male vs. female)	+ 4.40	2.97	−1.49 to + 10.30	0.143
*Diabetes duration (years)*	+ 0.38	0.15	+ 0.07 to + 0.68	**0.015**
BMI (kg/m²)	+ 0.35	0.25	−0.15 to + 0.86	0.172
Smoking (yes vs. no)	+ 1.94	2.40	−2.84 to + 6.71	0.423
Fasting plasma glucose (mg/dL)	+ 0.01	0.02	−0.02 to + 0.05	0.472
Hemoglobin (g/dL)	−2.36	2.72	−7.76 to + 3.03	0.387
Haematocrit (%)	−0.27	0.83	−1.91 to + 1.37	0.744

R² = 0.622; Adjusted R² = 0.585; *n* = 101; F-test overall *p* < 0.001

Outcome variable: Δ(HbA1c) = eMPG(HbA1c) − Home-CBG (mg/dL). Negative values indicate HbA1c underestimation

*SE* standard error; *CI* confidence interval; *BMI* body mass index

Bold values indicate statistical significance (p < 0.05)

## Conclusion

HbA1c systematically underestimates glycaemic status in patients with secondary polycythaemia, independent of major confounders, with a mean bias of approximately − 23 mg/dL sufficient to cause clinically relevant glycaemic misclassification in 60% of affected patients. In contrast, fructosamine showed closer agreement with real-life glucose measurements and appears to be a useful complementary biomarker when HbA1c is discordant with clinical findings. These results are particularly relevant in settings where altitude- and smoking-related erythrocytosis are prevalent. Larger prospective and multicentre studies incorporating CGM, erythrocyte lifespan measurement, and albumin-adjusted fructosamine are needed to confirm these findings and to define the role of fructosamine in routine clinical practice.

## Supplementary Information

Below is the link to the electronic supplementary material.


Supplementary Material 1

