# Supplementary material for: Glycemic assessment in diabetic patients with secondary polycytheamia: limitations of HbA1c and the potential role of fructosamine—a prospective comparative study
Source: Acta Diabetol. 2026 Jun 22;63(9):1545–52. doi: 10.1007/s00592-026-02735-z (PMC13534165; doi:10.1007/s00592-026-02735-z)
Supplement: Supplementary file 1 [file 592_2026_2735_MOESM1_ESM.docx]

Suplement

Table 6: Some laboratory parameters of the groups

|  | Polycythemia group  (N:62) | Control group  (N:40) |  |
| --- | --- | --- | --- |
|  | Median ± SD | Median ± SD | *p-value* |
| Total cholesterol, mg/dL | 191.97 **±** 58.59 | 183.23 **±** 32.62 | .795 |
| LDL cholesterol, mg/dL | 108.67 **±** 44.54 | 106.75 **±** 30.15 | .913 |
| HDL cholesterol, mg/dL | 41.20 **±** 7.99 | 42.88 **±** 10.21 | .444 |
| Triglycerides, mg/dL | 220.17 **±** 161.70 | 170.05 **±** 75.94 | .161 |
| Blood urea nitrogen, mg/dL | 14.82 **±** 3.44 | 16.22 **±** 6.68 | .600 |
| Creatinine, mg/dL | 0.82 **±** 0.15 | 0.79 **±** 0.19 | .340 |
| Uric acid, mg/dL | 5.07 **±** 1.30 | 5.03 **±** 0.92 | .729 |
| *Red blood cells x10^9^/L* | 5.76 **±** 0.36 | 4.84 **±** 0.34 | ***.001*** |
| *Hemoglobin, g/dL* | 16.86 **±** 0.78 | 13.92 **±** 0.86 | ***.001*** |
| *Hematocrit, %* | 49.15 **±** 2.21 | 41.21 **±** 2.75 | ***.001*** |
| Mean corpuscular volume, fL | 84.95 **±** 3.29 | 84.89 **±** 3.55 | .752 |
| White blood cells x10^9^/L | 8.52 **±** 1.89 | 7.94 **±** 1.95 | .128 |
| Lymphocytes x10^9^/L | 3.17 **±** 4.13 | 2.67 **±** 0.78 | .872 |
| Neutrophils x10^9^/L | 4.99 **±** 1.60 | 4.47 **±** 1.30 | .132 |
| Platelets x10^9^/L | 246.13 **±** 58.70 | 265.38 **±** 56.42 | .051 |
| Mean platelet volume. fL | 10.45 **±** 0.89 | 10.37 **±** 0.76 | .692 |

Mann-Whitney U test

Abbreviations: LDL: Low- density cholesterol; HDL: High-density cholesterol
